# Wounds of the Chest and Abdomen

**Published:** 1829-05

**Authors:** 


### WOUNDS OF THE CHEST AND ABDOMEN.

*Wounds of the Chest and Abdomen, for the purpose of Suicide; Recovery.*

Persons who attempt to commit suicide are frequently brought to the Hôtel Dieu, where a portion of one of the wards (Saint-Bernard) is set apart for them.

Case I.—An individual was admitted February 26th, who had wounded himself by inflicting two stabs in the left side of the chest. His actions were controlled; the wounds (from which the blood flowed freely) were brought together, and he was bled in the arm. In half an hour, the difficulty of breathing having increased, a second venesection was employed, and a cupping glass applied over the wounds, by which means a large quantity of blood was removed. The wounds were again closed, and the patient was brought to the hospital within a few hours after the receipt of the injury. He was about forty-six years of age, a tailor, of middle stature, and rather robust, but without any thing in his countenance indicative of remarkable energy. On his admission, he was in a state of depression; the pulse feeble; almost the entire left side of the chest painful on pressure, but without any emphysema or obvious effusion of blood; the respiration was slow but regular, without cough or spitting: in short, there was no evidence of the lungs having been wounded. The patient became calm, and by the 10th of March (when the report is dated) the recovery was almost complete.

Case II.—A man, aged fifty-six, large and robust, having been arrested at the moment when he had stolen some money, seized a knife, placed the handle of it against the wall, and the point towards the epigastrium, and threw himself upon it. He fell. A practitioner was called, who brought the lips of the wound together, and bled him from the arm: two hours after which he was received at the Hôtel Dieu.

The skin was uniformly yellow, the eye haggard, and the countenance animated. The wound was transverse, about six lines in breadth, and, according to the practitioner who had first seen the patient, it was from an inch and a half to two inches in depth. There was nausea, but no vomiting; the pulse quick and hard; the entire circumference of the wound extremely tender to the touch. In the evening, as this last symptom continued, fifteen leeches were applied to the part; and in other respects the treatment consisted in diluents, cataplasms, and a strait-waistcoat. On the 10th the wound was nearly healed; but the pain continuing, the leeches were again applied; and once more on the 14th. On the 20th the patient was discharged, apparently hypochondriacal.

These cases are related, and others are referred to, with a view of showing that persons who attempt to commit suicide in consequence of some sudden impulse, as after the perpetration of a crime, seldom have resolution or coolness enough to secure the accomplishment of their object.—*La Clinique.*

